# AWGC2023 cachexia consensus as a valuable tool for predicting prognosis and burden in Chinese patients with cancer

**DOI:** 10.1002/jcsm.13555

**Published:** 2024-08-27

**Authors:** Hailun Xie, Lishuang Wei, Guotian Ruan, Heyang Zhang, Jinyu Shi, Shiqi Lin, Chenan Liu, Xiaoyue Liu, Xin Zheng, Yue Chen, Junqiang Chen, Hanping Shi

**Affiliations:** ^1^ Department of Gastrointestinal Gland Surgery, First Affiliated Hospital Guangxi Medical University Nanning China; ^2^ Department of Gastrointestinal Surgery, Department of Clinical Nutrition Beijing Shijitan Hospital, Capital Medical University Beijing China; ^3^ National Clinical Research Center for Geriatric Diseases, Xuanwu Hospital Capital Medical University Beijing China; ^4^ Key Laboratory of Cancer FSMP for State Market Regulation Beijing China

**Keywords:** Cancer cachexia, AWGC2023 criteria, Survival, Medical burden

## Abstract

**Background:**

The Asian Working Group for Cachexia (AWGC) proposed the first consensus report on diagnostic criteria for cachexia in Asians in 2023. However, the current consensus lacks cohort evidence to validate its effectiveness and practicality. We aimed to explore the value of the AWGC2023 criteria for predicting the prognosis and medical burden of patients with cancer through a retrospective post hoc cross‐sectional analysis of the Investigation on Nutrition Status and its Clinical Outcome of Common Cancers (INSCOC) project in China.

**Methods:**

Cox regression analyses were performed to assess the independent association between cachexia and long‐term survival. We utilized C‐reactive protein (CRP), neutrophil‐to‐lymphocyte ratio (NLR), inflammatory burden index (IBI), albumin (ALB) and Glasgow prognostic score (GPS) as diagnostic markers for cachexia, designating them as CRP‐based cachexia, NLR‐based cachexia, IBI‐based cachexia, ALB‐based cachexia and GPS‐based cachexia, respectively. Additionally, we diagnosed cachexia using body mass index (BMI) cutoff values of 18.5, 20, 21 and 22 kg/m^2^, respectively, and subsequently compared their prognostic predictive value through Harrell's concordance index (C‐index). Logistic regression models were used to assess the association between cachexia and medical burden.

**Results:**

A total of 5426 patients with cancer were enrolled in this study. Cox regression analysis confirmed that cachexia based on the AWGC2023 criteria was an independent predictor of long‐term survival in patients with cancer. Patients with cachexia had significantly poorer long‐term survival than patients without cachexia (66.4% vs. 49.7%, P < 0.001). Inflammatory biomarker‐based cachexia was as an independent predictor of prognosis in patients with cancer, with inflammatory burden index (IBI)‐based cachexia demonstrating the optimal prognostic discriminatory ability. The C‐index indicated that cachexia based on BMI cutoff values of 18.5, 20, and 22 kg/m^2^ did not perform as well as a BMI cutoff value of 21 kg/m^2^. Logistic regression models revealed that using the AWGC2023 criteria, patients with cachexia had a 16.6% higher risk of prolonged hospitalization and a 16.0% higher risk of high medical expenses than patients without cachexia.

**Conclusion:**

The AWGC2023 criteria represent a valuable tool for predicting survival and medical burden among Chinese patients with cancer. Encouragement for further validation in other Asian populations is warranted for the AWGC2023 criteria.

## Introduction

Cachexia is characterized by persistent skeletal muscle wasting and may be accompanied by loss of adipose tissue. Even with conventional nutritional therapy, symptoms cannot be completely relieved, ultimately leading to progressive functional impairment.^1^, ^2^ Cachexia is most common in patients with cancer, especially those with advanced malignancies, with approximately 60% to 80% of patients with advanced cancer developing cachexia.^3^, ^4^, ^5^ In patients with advanced cancer, cachexia can exacerbate the toxic side effects of chemotherapy, shorten its duration, reduce responsiveness to treatment, reduce quality of life, increase the incidence of complications and further increase mortality rates. Up to 22% of patients with cancer die from cachexia.^6^, ^7^ Therefore, timely identification of cachexia is essential for patients with cancer. Early detection and intervention can improve nutritional status, enhance treatment effectiveness, alleviate adverse reactions and improve quality of life. Therefore, medical institutions and healthcare providers should pay attention to screening and management of cachexia to help patients with cancer receive better care and treatment.

Currently, there are challenges in understanding and diagnosing cachexia. In clinical practice, the incidence and risk of cachexia are often underestimated.^8^, ^9^ One reason for this is the lack of public awareness and understanding of cachexia. The limited knowledge of healthcare professionals involved in cancer‐related work contributes to the insufficient recognition and treatment of cancer cachexia. A recent international survey revealed that oncology healthcare providers have a relatively limited understanding of cancer cachexia and its management. This can be attributed to a lack of widespread education and awareness campaigns.^10^ In addition, current international standards for diagnosing cachexia focus on the loss of body composition and muscle mass and require specific equipment for measurement, such as dual‐energy X‐ray absorptiometry, bioelectrical impedance analysis and computed tomography. These detection methods are both costly and complex, limiting their application in clinical practice.^2^, ^11^ Furthermore, existing international standards may underestimate the occurrence of cachexia in Asian populations. Asians have differences in physique compared with Westerners, with a lower mean body mass index (BMI) and differences in muscle mass and function.^12^, ^13^ Taking the above considerations into account, the Asian Working Group for Cachexia (AWGC) proposed the first consensus report on the diagnostic criteria and clinical outcomes of cachexia in Asian populations in 2023, to promote further research and use of cachexia in clinical practice in the Asian population.^14^

The consensus removed reduced muscle mass as a diagnostic criterion, added the measurement of hand‐grip strength (HGS) and proposed a BMI threshold of 21 kg/m^2^ for Asian populations. In addition, unlike the 2011 Cachexia Consensus, it also included inflammatory markers as a diagnostic criterion. However, the AWGC2023 criteria for cachexia lacks cohort evidence that it predicts survival. Many inflammatory markers have been shown to effectively predict cancer cachexia and adverse outcomes. These include the inflammatory burden index (IBI), neutrophil‐to‐lymphocyte ratio (NLR), and Glasgow prognostic score (GPS), and further evaluations of these inflammatory markers for cachexia diagnosis are needed.^15^, ^16^ In addition, there is a lack of consistency regarding the BMI threshold for diagnosing cancer cachexia in Asian populations.

Therefore, we conducted a multicentre cohort study in the Chinese population to explore the value of the AWGC2023 criteria for predicting the prognosis and medical burden of patients with cancer and compared the prognostic value of cachexia defined based on different inflammatory markers. In addition, we compared the diagnostic accuracy of different BMI cutoff values for diagnosing cachexia. This study aimed to provide reliable cohort evidence of the effectiveness and practicality of the AWGC2023 criteria for cachexia.

## Materials and methods

### Population

All patients were recruited from the Investigation on Nutrition Status and its Clinical Outcome of Common Cancers (INSCOC) project, which recruited participants from multiple clinical centres across China in 2013. The INSCOC project was registered in the Chinese Clinical Trial Registry (ChiCTR1800020329, http://www.chictr.org.cn) The design and methods of the INSCOC project have been described previously.^17^, ^18^ This study is a retrospective post hoc cross‐sectional analysis of participants from the INSCOC project with histologically or cytologically confirmed cancer and whose peripheral blood cell data and follow‐up information were complete. To ensure the accuracy and reliability of the research results, we excluded the following patients from the analysis: those with multiple types of tumours, those with obvious systemic infection or inflammatory symptoms, those under 18 years of age, and those with severe conditions such as heart failure and renal dysfunction. The study was performed in accordance with the ethical standards laid down in the 1964 Declaration of Helsinki and its later amendments. The study protocol was approved by the ethics committees of the participating institutions, and all patients provided written informed consent. The data were de‐identified to protect patient privacy and personal information security. All personal identifiers were removed before analysing the data.

### Clinicopathological variables

The clinicopathological variables of the patients were collected prior to any treatment. The demographic information included sex, age, comorbidities (hypertension, diabetes and coronary heart disease), smoking history and alcohol consumption. Clinical information collected included self‐reported decreased food intake, family cancer history, weight loss (at least 3 months of weight recall), pathology information (stage and tumour type), antineoplastic treatment (surgery, radiotherapy and chemotherapy), length of hospital stay and hospital expenses. Recent nutritional and physical activity information at baseline, including Patient‐Generated Subjective Global Assessment (PG‐SGA) score, Eastern Cooperative Oncology Group (ECOG) score and quality of life. Quality of life information were collected on the day of admission using the European Organization for Research and Treatment of Cancer Quality of Life Questionnaire (EORTC QLQ‐C30 Version 3.0, QoL). Anthropometric measurements, including height, body weight, HGS and BMI, were also obtained. BMI is calculated by weight (kg)/height (m)^2^. The HGS was measured by an electronic handgrip dynamometer (CAMRY, Model EH101, Guangdong, China). Patients were asked to stand comfortably, then to perform three maximal isometric contractions 30 s apart using their dominant hand. The maximal read for the handgrip strength was recorded. Data were collected on haematology and blood biochemistry, including white blood cell, neutrophil, lymphocyte, platelet, red blood cell counts, haemoglobin, C‐reactive protein (CRP) and albumin (ALB) levels. Serum data were obtained through peripheral venous puncture and blood testing within 48 h of admission. In this study, we used previously reported serum inflammatory markers to evaluate inflammatory burden, including CRP, NLR, IBI, ALB and GPS. The NLR was defined as the ratio of neutrophils to lymphocytes. IBI was defined as the product of CRP level (mg/dL), neutrophil count (10^9^/L), and lymphocyte count (10^9^/L). The GPS was scored as follows: CRP ≤ 10 mg/L and ALB ≥ 35 g/L: 0; CRP ≤ 10 mg/L or ALB < 35 g/L: 1; and CRP > 10 mg/L and ALB < 35 g/L: 2. Standardized log‐rank statistics were used to determine the optimal cutoff values for NLR, IBI and ALB, which were 3.4, 16, and 37 g/L, respectively (*Figure* *S1*).

All patients in the INSCOC cohort are hospitalized with a confirmed cancer diagnosis requiring further treatment, and they were recruited upon admission. The primary outcome of this study was long‐term survival, defined as the interval from patient admission to death or the last follow‐up date. Secondary outcomes of the study included short‐term outcomes, defined as the mortality of patients within 3 months after admission, prolonged length of hospital stay (≥14 days) and hospital expenses ≥20 000 yuan.

### 2011 Cachexia Consensus and AWGC2023 criteria for cachexia

The 2011 Cachexia Consensus defines cachexia based on the following criteria^11^: (1) unintentional weight loss of >5% within 6 months without dieting; (2) BMI < 18.5 kg/m^2^ and any degree of weight loss >2%; (3) appendicular skeletal muscle index meeting the criteria for muscle depletion (men <7.26 kg/m^2^, women <5.45 kg/m^2^), and any degree of weight loss >2%.

The AWGC2023 criteria defines cachexia based on the following criteria^14^: (1) presence of an underlying disease, including cancer; (2) weight loss >2% in 3–6 months or low BMI (<21 kg/m^2^); (3) one or more of the following: (i) subjective symptoms: anorexia; (ii) objective measurement: decreased HGS (<28 kg in men and <18 kg in women); (iii) inflammatory biomarkers: elevated CRP level (>5 mg/L). In this study, we used different inflammatory markers (CRP level, NLR, IBI, ALB level and GPS) to define the inflammatory biomarker criterion of the AWGC2023 criteria for cachexia. Additionally, we compared the effects of using different BMI cutoff values (18.5, 20, 21 and 22 kg/m^2^) to define cachexia according to the AWGC2023 criteria.

### Study design

**Figure 1:**
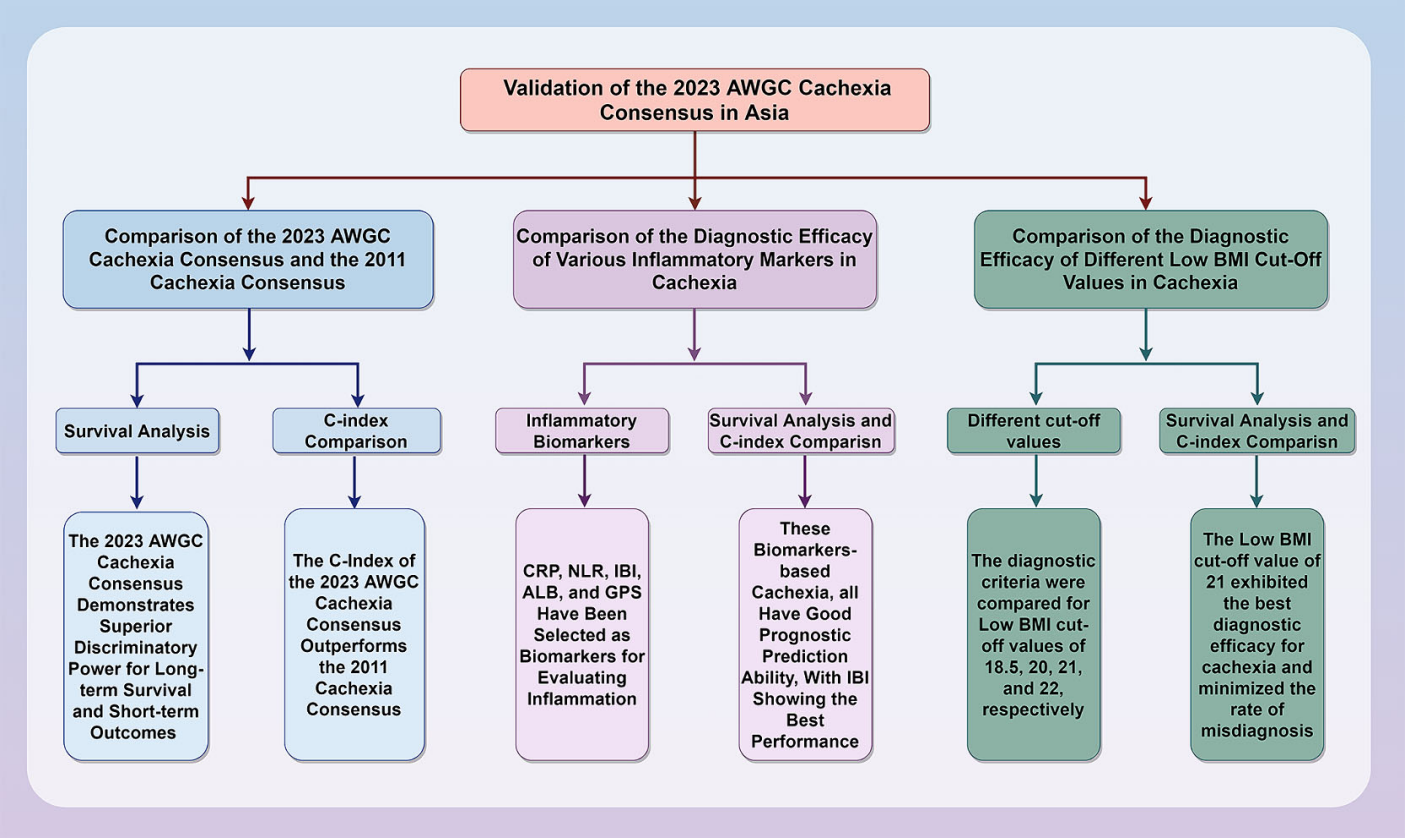
Study design.

The study design is depicted in *Figure* *1*. Initially, we validated the prognostic value of the AWGC2023 criteria for cachexia in the Chinese cancer population. Subsequently, we compared the performance of the AWGC2023 criteria and 2011 Cachexia Consensus definitions of cachexia for predicting the long‐term survival of patients with cancer. Inflammatory biomarkers play a crucial role in the AWGC2023 criteria. However, there is still a lack of evidence to determine the optimal inflammatory biomarker for the diagnosis of cachexia. Therefore, we diagnosed cachexia using CRP, NLR, IBI, ALB and GPS, labelling them as CRP‐based cachexia, NLR‐based cachexia, IBI‐based cachexia, ALB‐based cachexia and GPS‐based cachexia, respectively. We then proceeded to compare their prognostic value. The BMI cutoff values for cachexia remain controversial. The 2011 Cachexia Consensus used a cutoff value of 18.5 kg/m^2^ whereas the AWGC2023 criteria adopted a value of 21 kg/m^2^. Given this disparity, it is imperative to assess various BMI cutoff values to determine the most suitable BMI threshold for cachexia diagnosis. Consequently, we diagnosed cachexia using BMI cutoff values of 18.5, 20, 21 and 22 kg/m^2^ and subsequently compared their prognostic predictive value.

### Statistical analysis

Continuous data were reported as the mean and standard deviation (*SD*) or median and interquartile range (IQR) whereas categorical data were reported as frequencies and percentages. Student's *t* test was used to analyse intergroup differences in continuous variables. Chi‐square tests or Fisher's exact test were used to compare categorical variables. Survival curves were plotted using the Kaplan–Meier method. The log‐rank test was used to compare survival rates, and the chi‐square test was used to compare differences in other characteristics between groups. Cox regression analyses were performed to assess the independent association between cachexia and long‐term survival. Sensitivity analyses were performed by excluding patients who died within 3, 6 and 12 months to evaluate the robustness of the results. Furthermore, a subgroup analysis was conducted of different tumour subgroups to test the relationship between cachexia and outcomes in different tumour types. Harrell's concordance index (C‐index) was used to evaluate the discrimination performance of different cachexia consensus definitions for predicting survival. Logistic regression models were used to assess the association between the AWGC2023 criteria and short‐term outcomes, length of hospital stay and hospital expenses. All statistical analyses were performed using IBM SPSS (Version 26.0; IBM Corp, Armonk, NY, USA). Two‐sided *P* values <0.05 were considered statistically significant.

## Results

### Baseline characteristics

This study included 5426 patients, of whom 3268 (60.2%) were male. *Figure* *S2* shows patient inclusion and exclusion flow chart. The mean age was 59.31 ± 11.20 years. Patients' cancer was classified into stages I to IV, with 10.4%, 19.7%, 26.0% and 43.9% of the patients having stage I, II, II and IV cancer, respectively. The most common primary cancer sites were the lung and bronchus (33.1%), colon and rectum (19.8%) and gastric (14.7%) regions. According to the AWGC2023 criteria, 2374 patients (43.8%) had cachexia. The prevalence of cachexia differed according to the tumour type. Patients with pancreatic cancer had the highest prevalence of cachexia (68.9%), followed by gastric cancer (57.7%) and oesophageal cancer (54.0%). Patients with urinary system tumours (29.6%) and breast cancer (18.6%) had a lower prevalence of cachexia than patients with digestive system tumours (*Figure* *S3*).

Patients with cachexia had higher rates of prolonged length of hospital stay (*P* < 0.001), inflammation (CRP, *P* < 0.001), poor nutrition (PG‐SGA, *P* < 0.001), reduced quality of life (*P* < 0.001) and increased hospital expenses (*P* < 0.001). Additionally, patients with cachexia were more likely to be male (*P* < 0.001), older (*P* < 0.001) and have a low BMI (*P* < 0.001) and advanced cancer (*P* < 0.001). Patients with cachexia had 7.4% and 16.7% higher short‐ and long‐term mortality rates, respectively, than patients without cachexia (all *P* < 0.001). Conversely, patients without cachexia were more likely to undergo surgery or chemotherapy for cancer treatment (all *P* < 0.001) (*Table* *S1*).

### Association between the cachexia and survival

**Figure 2:**
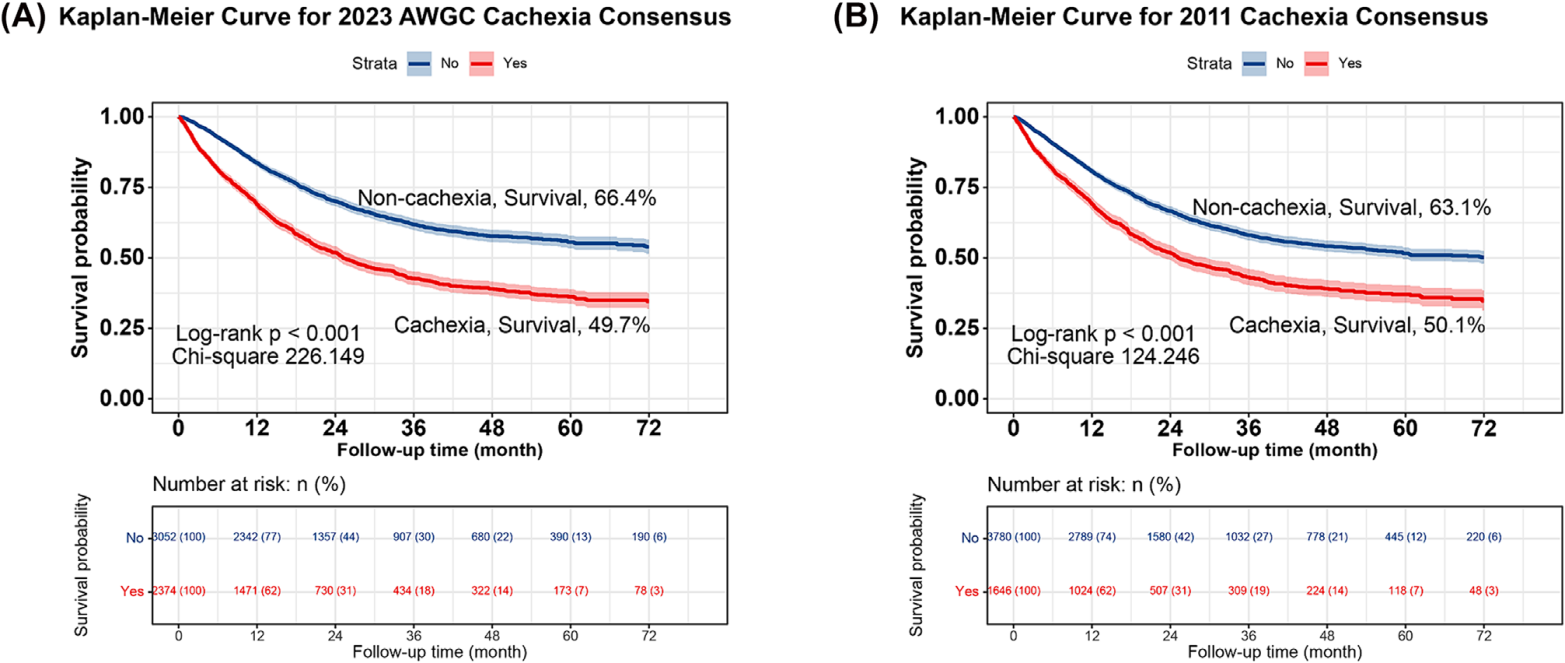
Comparison of the AWGC2023 criteria (*A*) and the 2011 Cachexia Consensus (*B*). AWGC, Asian Working Group for Cachexia.

Kaplan–Meier survival curves demonstrated that cachexia patients had significantly poorer long‐term survival than patients without cachexia (66.4% vs. 49.7%, *P* < 0.001) (*Figure* *2* *A*). Additionally, the subgroup analysis revealed that patients with cachexia had significantly worse survival across each pathological stage than that of patients without cachexia (*Figure* *S4*, all *P* < 0.001). Notably, cachexia provides the most effective prognostic differentiation in stage IV cancer, surpassing its effectiveness in other pathological stages (25.880 vs. 42.816 vs. 29.502 vs. 61.657). After adjusting for confounding factors such as age, sex, BMI, TNM stage, tumour type, surgery, radiotherapy, chemotherapy, hypertension, diabetes, coronary heart disease, smoking, drinking and family history, Cox regression analysis revealed that cachexia serves as an independent predictor of long‐term survival in patients with cancer (hazard ratio [HR]: 1.458, 95% confidence interval [CI]: 1.328–1.601, *P* < 0.001) (*Table* *1*). Forest plots of survival for different tumour types revealed that cachexia was an independent risk factor for predicting long‐term survival in most tumour types, including lung and bronchus, oesophagus, gastric, liver and intrahepatic bile duct, colon and rectum, breast, gynaecological, urological and nasopharyngeal cancers (*Figure* *S5*).

**Table 1:** Cox regression analysis of the AWGC2023 criteria and the 2011 Cachexia Consensus.

Categories	Model a (HR, 95% CI)	*P* value	Model b (HR, 95% CI)	*P* value	Model c (HR, 95% CI)	*P* value
AWGC2023 criteria	1.881 (1.730,2.045)	<0.001	1.483 (1.352,1.628)	<0.001	1.458 (1.328,1.601)	<0.001
2011 Cachexia Consensus	1.626 (1.491,1.772)	<0.001	1.376 (1.256,1.508)	<0.001	1.391 (1.269,1.526)	<0.001

*Note*: Model a: Not adjusted. Model b: Adjusted for age, sex, body mass index (BMI), TNM stage. Model c: Adjusted for age, sex, BMI, TNM stage, tumour type, surgery, radiotherapy, chemotherapy, hypertension, diabetes, coronary heart disease, smoking, drinking and family history.

Abbreviations: AWGC, Asian Working Group for Cachexia; CI, confidence interval; HR, hazard ratio.

### Comparison of AWGC2023 criteria and 2011 Cachexia Consensus definitions of cachexia for predicting survival

The Kaplan–Meier survival curves showed that both the AWGC2023 criteria (*Figure* *2* *A*) and the 2011 Cachexia Consensus (*Figure* *2* *B*) definitions of cachexia could effectively stratify the long‐term survival of patients with cancer. However, the AWGC2023 criteria had better prognostic discrimination than that of the 2011 Cachexia Consensus (with chi‐square values of 226.149 and 124.246, respectively). Using the AWGC2023 criteria, patients with cachexia had a 45.8% increased overall risk of death compared with patients without cachexia (HR: 1.458, 95% CI: 1.328–1.601, *P* < 0.001). Using the 2011 Cachexia Consensus definition, patients with cachexia had a 39.1% increased overall risk of death compared with patients without cachexia (HR: 1.391, 95% CI: 1.269–1.526, *P* < 0.001) (*Table* *1*). Prognostic stratification of the AWGC2023 criteria was better than that of the 2011 Cachexia Consensus criteria. Sensitivity analysis showed the robustness of the results when deaths within 3, 6 and 12 months of follow‐up were excluded (*Table* *S2*). Furthermore, we compared the discriminative abilities of different cachexia definitions in predicting the prognosis using the C‐index. The AWGC2023 criteria showed a 2.8% improvement in the predictive prognostic value for patients with cancer compared with the 2011 Cachexia Consensus (−0.028, 95% CI: −0.038 to −0.018, *P* < 0.001) (*Table* *2*). Additionally, we used a Venn diagram to show the intersection of cachexia diagnoses between the AWGC2023 criteria and the 2011 Cachexia Consensus. The results indicated that the AWGC2023 criteria included most cases diagnosed by the 2011 Cachexia Consensus criteria but identified additional cases of cachexia not identified by the 2011 Cachexia Consensus definition (*Figure* *S6*). These results suggest that cachexia defined according to the AWGC2023 criteria is superior to that defined according to the 2011 Cachexia Consensus criteria for predicting long‐term survival of patients with cancer in China and can reduce cachexia misdiagnosis rates.

**Table 2:** The C‐index of the AWGC2023 criteria and the 2011 Cachexia Consensus.

Discrimination ability	C‐index		
	Difference	Difference	*P* value
AWGC2023 criteria	0.587 (0.576, 0.598)	Ref	
2011cachexia consensus	0.559 (0.548, 0.570)	−0.028 (−0.038, −0.018)	<0.001

Abbreviations: AWGC, Asian Working Group for Cachexia.

### Evaluation of the effectiveness of inflammatory markers for diagnosing cachexia

**Figure 3:**
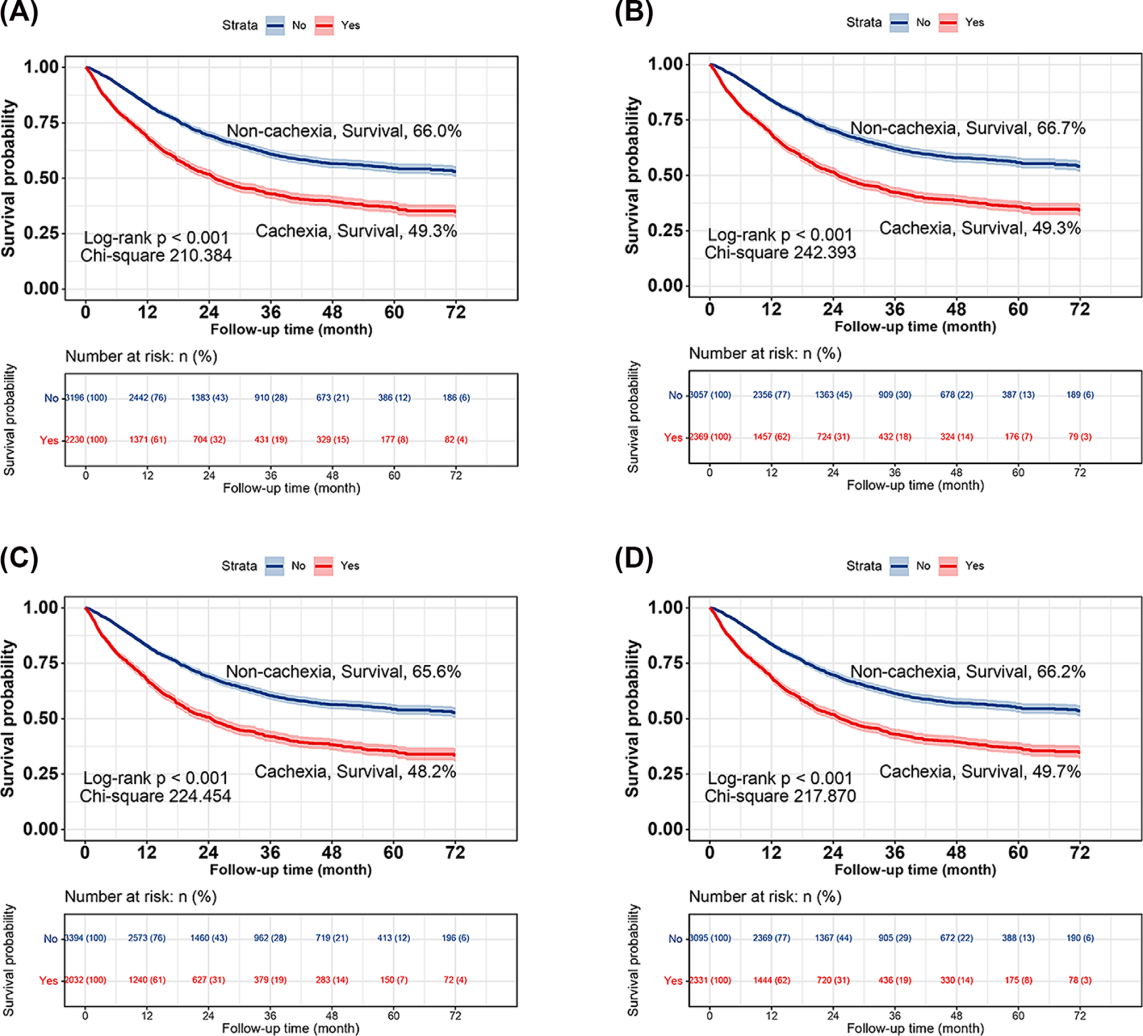
Comparison of the diagnostic efficacy of various inflammatory markers in cachexia. Kaplan–Meier curves for (*A*), NLR‐based cachexia; (*B*), IBI‐based cachexia; (*C*), ALB‐based cachexia; (*D*), GPS‐based cachexia. ALB, albumin; CRP, C‐reactive protein; GPS, Glasgow prognostic score; IBI, inflammatory burden index; NLR, neutrophil‐to‐lymphocyte ratio.

2374 (43.8%) patients were diagnosed with CRP‐based cachexia, 2230 (41.1%) patients were diagnosed with NLR‐based cachexia, 2369 (41.1%) patients were diagnosed with IBI‐based cachexia, 2032 (43.7%) patients were diagnosed with ALB‐based cachexia, and 2331 (43.0%) patients were diagnosed with GPS‐based cachexia. The Kaplan–Meier survival curves showed that all these inflammation biomarker‐based cachexia definitions predicted survival in patients with cancer (*Figure* *3*). Cox regression analysis revealed that inflammatory biomarker‐based cachexia was an independent risk factor affecting the prognosis of patients with cancer (*Table* *3*). A sensitivity analysis that excluded patients with short‐term mortality demonstrated the robustness of inflammatory biomarker‐based cachexia in predicting the prognosis of patients with cancer (*Table* *S3*). By comparing their chi‐square values, we found that IBI showed the best prognostic discriminatory ability, followed by CRP, ALB, GPS and NLR, with chi‐square values of 242.393, 226.149, 224.454, 217.870 and 210.384, respectively. Next, we compared the C‐index of the inflammatory biomarker‐based cachexia types. Using CRP‐based cachexia as a reference, only the IBI‐based cachexia definition demonstrated positive clinical benefits (HR: 0.003, 95% CI: 0.005–0.006, *P* = 0.024) (*Table* *4*). The Venn diagram showed considerable overlap in the diagnostic ability of these inflammatory biomarker‐based cachexia definitions, reaching 83.9% (1992 cases), with IBI‐based and ALB‐based cachexia having the highest overlap with other inflammatory biomarker‐based cachexia definitions. This suggests that most inflammatory biomarker‐based cachexia definitions have good diagnostic capabilities, with IBI‐based and ALB‐based cachexia definitions showing high specificity (*Figure* *S7*).

**Table 3:** Cox regression analysis of the inflammatory markers‐based cachexia.

Categories	Model a (HR, 95% CI)	*P* value	Model b (HR, 95% CI)	*P* value	Model c (HR, 95% CI)	*P* value
NLR‐based cachexia	1.836 (1.689, 1.996)	<0.001	1.489 (1.356, 1.634)	<0.001	1.481 (1.348, 1.627)	<0.001
IBI‐based cachexia	1.922 (1.768, 2.09)	<0.001	1.534 (1.397, 1.683)	<0.001	1.504 (1.37, 1.652)	<0.001
ALB‐based cachexia	1.874 (1.724, 2.037)	<0.001	1.525 (1.389, 1.675)	<0.001	1.526 (1.39, 1.676)	<0.001
GPS‐based cachexia	1.858 (1.709, 2.019)	<0.001	1.493 (1.36, 1.638)	<0.001	1.473 (1.341, 1.619)	<0.001

*Note*: Model a: Not adjusted. Model b: Adjusted for age, sex, BMI, TNM stage. Model c: Adjusted for age, sex, BMI, TNM stage, tumour type, surgery, radiotherapy, chemotherapy, hypertension, diabetes, coronary heart disease, smoking, drinking and family history.

Abbreviations: ALB, albumin; CI, confidence interval; GPS, Glasgow prognostic score; HR, hazard ratio; IBI, inflammatory burden index; NLR, neutrophil‐to‐lymphocyte ratio.

**Table 4:** Comparison of the C‐index of the inflammatory markers‐based cachexia.

Discrimination ability	C‐index		
	Difference	Difference	*P* value
CRP‐based cachexia	0.587 (0.576, 0.598)	Ref	
NLR‐based cachexia	0.584 (0.574, 0.595)	−0.002 (−0.007, 0.004)	0.512
IBI‐based cachexia	0.590 (0.579, 0.601)	0.003 (0.005, 0.006)	0.024
ALB‐based cachexia	0.585 (0.574, 0.596)	−0.002 (−0.008, 0.004)	0.525
GPS‐based cachexia	0.587 (0.576, 0.597)	−0.000 (−0.004, 0.003)	0.907

Abbreviations: ALB, albumin; CRP, C‐reactive protein; GPS, Glasgow prognostic score; IBI, inflammatory burden index; NLR, neutrophil‐to‐lymphocyte ratio.

### Effectiveness of different BMI cutoff values for diagnosing cachexia

**Figure 4:**
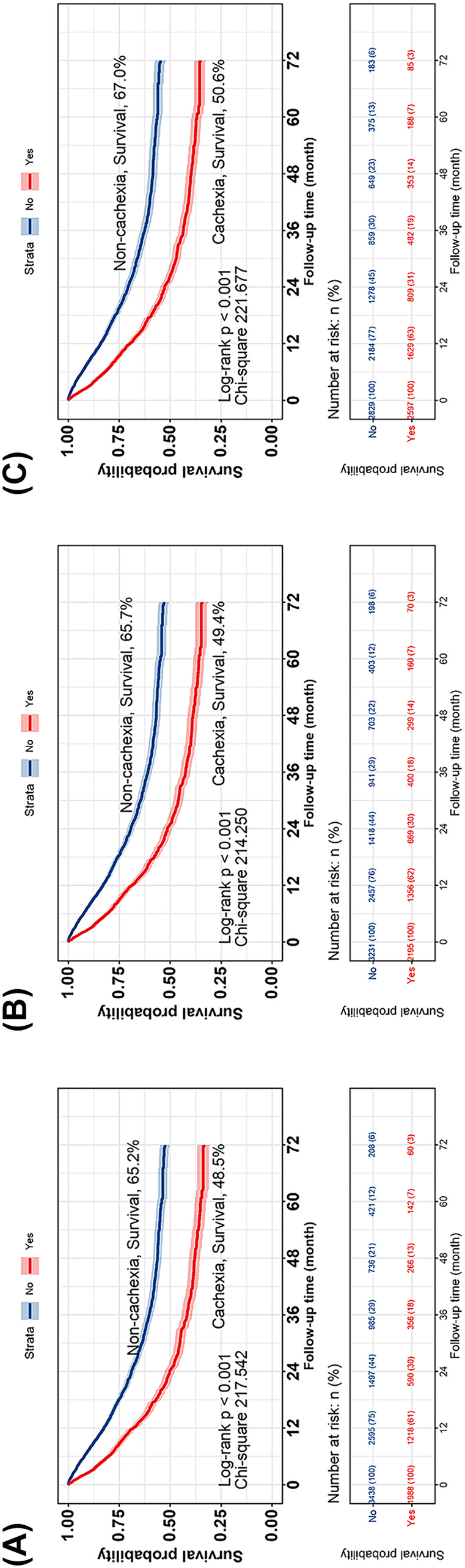
Comparison of the diagnostic efficacy of different low BMI cut‐off values in cachexia. Kaplan–Meier curves for (*A*), BMI < 18.5; (*B*), BMI < 20; (*C*), BMI < 22. BMI, body mass index.

Cox regression analysis revealed that cachexia based on different BMI cutoff values was an independent risk factor influencing the prognosis of patients with cancer (*Table* *S4*). Kaplan–Meier survival analysis demonstrated that all these BMI cutoff value‐based cachexia effectively stratified patients for prognosis, albeit with some differences in discriminatory ability. The chi‐square values, in descending order, were 226.149, 221.677, 217.542 and 214.250 in patients with a BMI less than 21, 22, 18.5 and 20 kg/m^2^, respectively (*Figure* *4*). The C‐index results indicated that cachexia based on BMI cutoff values of 18.5, 20 and 22 kg/m^2^ did not show positive clinical benefits compared with cachexia based on a BMI cutoff of 21 kg/m^2^ (*Table* *S5*). These findings suggest that a BMI cutoff value of 21 kg/m^2^ is the optimal threshold for diagnosing cachexia.

### Association between cachexia and short‐term outcomes

We analysed the predictive ability of different cachexia criteria for predicting the short‐term outcome of patients with cancer using logistic regression. The results showed that all the cachexia criteria were effective tools for predicting adverse short‐term outcomes (*Table* *S6*). Notably, the prognostic stratification effect of the AWGC2023 criteria was significantly better than that of the 2011 Cachexia Consensus. According to the AWGC2023 criteria, the patients with cachexia had a 113.8% increased risk of adverse short‐term outcomes in (odds ratio [OR]: 2.138, 95% CI: 1.625–2.811, *P* < 0.001). According to the 2011 Cachexia Consensus, patients with cachexia had a 77.7% higher risk of adverse short‐term outcomes than patients without cachexia (OR: 1.777, 95% CI: 1.393–2.266, *P* < 0.001). Moreover, the stratification effect of different inflammatory biomarker‐based cachexia types on adverse short‐term outcomes was similar.

### Association between cachexia and medical burden

We also explored the relationship between cachexia and medical burden (length of stay and hospital expenses). When cachexia was diagnosed using the AWGC2023 criteria, patients with cachexia had a 16.6% increased risk of prolonged hospitalization compared with patients without cachexia (OR: 1.166, 95% CI: 1.025–1.326, *P* = 0.020) and a 16.0% increased risk of high medical expenses (OR: 1.160, 95% CI: 1.022–1.317, *P* = 0.022). In contrast, when cachexia was diagnosed using the 2011 Cachexia Consensus, it was not an independent risk factor for prolonged hospitalization (OR: 1.082, 95% CI: 0.949–1.234, *P* = 0.238) or high medical expenses (OR: 1.112, 95% CI: 0.977–1.265, *P* = 0.109). Additionally, most inflammation biomarker‐based cachexia biomarkers were identified as independent risk factors for prolonged hospitalization and high medical expenses (*Tables* *S7* and *S8*).

## Discussion

Cachexia, a severe but underrecognized condition, has a negative effect on cancer outcomes.^19^, ^20^ As a new diagnostic standard for cachexia in Asian populations, the AWGC2023 criteria still require extensive validation. In this study, our findings indicate that the AWGC2023 criteria are a simple and effective tool for assessing the prognosis of patients with cancer. Compared with the 2011 Cachexia Consensus, the AWGC2023 criteria have shown a satisfactory improvement in prognostic prediction. Additionally, our study provides new insights into the use of inflammatory markers within the AWGC2023 criteria, suggesting that IBI may be the optimal inflammatory biomarker in its inflammatory component. Furthermore, our research provides reliable evidence for setting a BMI threshold at 21 kg/m^2^.

In this study, we validated the effectiveness the AWGC2023 criteria as a diagnostic tool for cancer cachexia. Cancer cachexia significantly impacts the health and survival of patients. Patients with cachexia face a 45% higher risk of poor prognosis compared with those without cachexia. Cachexia is a severe complication in patients with cancer, and its incidence has been consistently underestimated. Our research revealed that cachexia is widespread among cancer patients, especially those with digestive system tumours. The distinct location of digestive system tumours, which greatly affects nutritional intake, contributes to a notably high prevalence of cachexia in these patients, exceeding that seen in other systemic tumours. Despite significant differences in the occurrence rates of cachexia among different types of tumours, the AWGC2023 criteria‐based cachexia remains an independent risk factor for poor prognosis in most tumour types. The high prevalence of cachexia in tumours not only causes physical and psychological harm to patients with cancer but also imposes a significant burden on the healthcare system. Arthur et al.^21^ showed that cachexia is strongly associated with increased hospitalization time, higher healthcare costs and greater functional impairment. Similarly, we identified cachexia as an important factor contributing to the increased risk of long hospital stays and higher medical expenses in patients with cancer. Short‐term mortality is another key criterion for assessing the effectiveness of a tool. We have found that the AWGC2023 criteria are an effective tool for evaluating short‐term mortality in patients with cancer.

The 2011 Cachexia Consensus is currently the most widely used diagnostic criteria for cancer cachexia, making it essential to compare the prognostic predictive efficacy of the AWGC2023 criteria with the 2011 Cachexia Consensus. Our findings indicate that, in comparison to the 2011 Cachexia Consensus, the AWGC2023 criteria demonstrated better performance in both the differentiation of Kaplan–Meier survival curves and the accuracy of prognosis prediction assessed by the C‐index. The AWGC2023 criteria removed the relatively complex diagnostic criteria for reduced muscle mass and replaced them with the simpler HGS, which has the advantage of being simple and easy to perform, especially suitable for primary healthcare units lacking muscle measurement tools. It is precisely due to this simplicity that the AWGC2023 criteria are poised to become a widely used diagnostic tool for cachexia, thereby aiding in the treatment and recovery of patients with cancer.

Inflammatory biomarkers are identified as important components in the AWGC2023 criteria. Although CRP is recommended for use, optimal inflammatory biomarkers require further investigation. We constructed an inflammatory biomarker‐based cachexia model using well‐established systemic inflammatory markers, such as CRP, NLR, ALB and GPS, based on previous studies, and compared their diagnostic performance. The results showed that inflammatory biomarker‐based cachexia could effectively predict adverse outcomes in patients with cancer, with IBI‐based cachexia demonstrating the best prognostic discrimination. This suggests that IBI may serve as the optimal inflammatory biomarker for the inflammatory component of AWGC2023 criteria. In clinical practice, especially in community hospitals, CRP is not a routine indicator due to its expensive and complex nature. On the other hand, the cost‐effective NLR and ALB, serving as inflammation markers are currently routine outpatient and inpatient laboratory tests. Furthermore, we have observed that NLR‐ and ALB‐based cachexia remains an effective tool in predicting adverse outcomes in patients with cancer. Compared with CRP‐based cachexia, their prognostic capabilities are remarkably similar. These findings suggest that when CRP testing tools are lacking, consideration can be given to NLR and ALB as affordable and widely available inflammatory markers in AWGC2023 criteria.

In the AWGC2023 criteria, the cutoff value for BMI is also an important component but remains controversial. Therefore, we diagnosed cachexia using BMI cutoff values of 18.5, 20, 21 and 22 kg/m^2^, respectively, and compared their predictive abilities for prognosis. The results showed that cachexia with a BMI cutoff of 21 kg/m^2^ had the highest prognostic stratification effect. As the global prevalence of obesity continues to rise, increasing the BMI threshold in the cachexia diagnostic criteria may be beneficial. Unlike the BMI threshold of 18.5 kg/m^2^, a BMI threshold of 21 kg/m^2^ can potentially identify a larger number of cachexia patients, particularly those with sarcopenic obesity. This study provides reliable evidence for setting a BMI threshold at 21 kg/m^2^.

This study validates the effectiveness of the AWGC2023 criteria in the Chinese cancer population and provides scientific evidence for the selection of inflammatory biomarker and the determination of BMI thresholds within the AWGC2023 criteria. However, there are still some limitations in this study that need to be clarified. First, the study population only included Chinese patients with cancer, which may have introduced a selection bias. Therefore, the effectiveness of the AWGC2023 criteria for cachexia needs to be validated in cancer cohorts from other Asian countries. Second, the study focused on patients with cancer; therefore, the generalizability of the results may not be generalizable to patients with other chronic diseases. Finally, although this was a multicentre study, an external validation cohort was not established. Therefore, future studies should validate these results in other populations.

## Conclusion

We confirmed the effectiveness of the AWGC2023 criteria for cachexia for predicting the prognosis and medical burden of patients with cancer. Compared with the 2011 Cachexia Consensus, this standard has better prognostic discrimination and predictive accuracy in Chinese patients with cancer. Additionally, the results provide evidence supporting the inclusion of inflammatory biomarkers in the AWGC2023 criteria for cachexia and provide evidence for setting a BMI cutoff value of 21 kg/m^2^.

## Conflicts of interest

The authors declare that they have no conflicts of interest.

## Funding

This study was supported by the Young Elite Scientists Sponsorship Program by CAST (2022QNRC001) and National Key Research and Development Program (No. 2017YFC1309200, No. 2022YFC2009600).

## Supporting information


**Figure S1.** The optimal cutoff values for inflammatory markers.
**Figure S2.** Patients flow chart.
**Figure S3.** The incidence of cachexia in different tumour types.
**Figure S4.** Kaplan–Meier curve of AWGC2023 criteria in patients with cancer at different pathological stage.
**Figure S5.** Subgroup Survival Forest Plot of Different Tumour Type.
**Figure S6.** The intersection of diagnosed cachexia cases between the AWGC2023 criteria and the 2011 Cachexia Consensus.
**Figure S7.** Venn Diagram of Various Inflammatory Markers‐based Cachexia.
**Table S1.** Clinicopathological characteristics.
**Table S2.** The sensitivity analysis by excluding the first 3, 6, 12 months mortalities of the AWGC2023 criteria and the 2011 Cachexia Consensus.
**Table S3**. The sensitivity analysis by excluding the first 3, 6, 12 months mortalities of the Inflammatory Markers‐based Cachexia.
**Table S4**. Cox regression analysis of the Different Low BMI Cut‐Off Value.
**Table S5**. Comparative analysis of the discrimination of the Different Low BMI Cut‐Off Value.
**Table S6**. Logistic regression analysis of the different cachexia criterias in predicting Short‐term outcome of patients with cancer.
**Table S7**. Logistic regression analysis of the different cachexia criterias in predicting length of stay (≥14 days) of patients with cancer.
**Table S8**. Logistic regression analysis of the different cachexia criterias in predicting expenses (≥20,000 yuan) of patients with cancer.

## Data Availability

The datasets used and/or analysed during the current study are available from the corresponding author on reasonable request.
