# Supplementary material for: AWGC2023 cachexia consensus as a valuable tool for predicting prognosis and burden in Chinese patients with cancer
Source: J Cachexia Sarcopenia Muscle. 2024 Aug 27;15(5):2084–93. doi: 10.1002/jcsm.13555 (PMC11446680; doi:10.1002/jcsm.13555)
Supplement: Supplementary file 1 — Figure S1. The optimal cutoff values for inflammatory markers. Figure S2. Patients flow chart. Figure S3. The incidence of cachexia in different tumour types. Figure S4. Kaplan–Meier curve of AWGC2023 criteria in patients with cancer at different pathological stage. Figure S5. Subgroup Survival Forest Plot of Different Tumour Type. Figure S6. The intersection of diagnosed cachexia cases between the AWGC2023 criteria and the 2011 Cachexia Consensus. Figure S7. Venn Diagram of Various Inflammatory Markers‐based Cachexia. Table S1. Clinicopathological characteristics. Table S2. The sensitivity analysis by excluding the first 3, 6, 12 months mortalities of the AWGC2023 criteria and the 2011 Cachexia Consensus. Table S3. The sensitivity analysis by excluding the first 3, 6, 12 months mortalities of the Inflammatory Markers‐based Cachexia. Table S4. Cox regression analysis of the Different Low BMI Cut‐Off Value. Table S5. Comparative analysis of the discrimination of the Different Low BMI Cut‐Off Value. Table S6. Logistic regression analysis of the different cachexia criterias in predicting Short‐term outcome of patients with cancer. Table S7. Logistic regression analysis of the different cachexia criterias in predicting length of stay (≥14 days) of patients with cancer. Table S8. Logistic regression analysis of the different cachexia criterias in predicting expenses (≥20,000 yuan) of patients with cancer. [file JCSM-15-2084-s001.docx]

**Figure S1.** The optimal cutoff values for inflammatory markers.

**
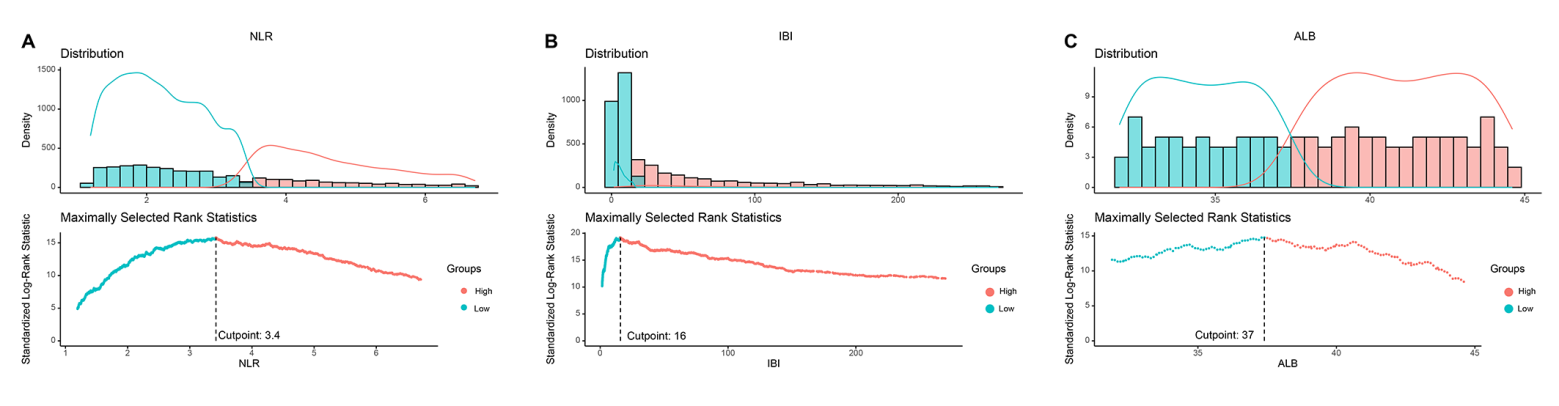
**

**Notes:** A, NLR; B, IBI; C, ALB.

**Figure S2.** Patients flow chart.

**
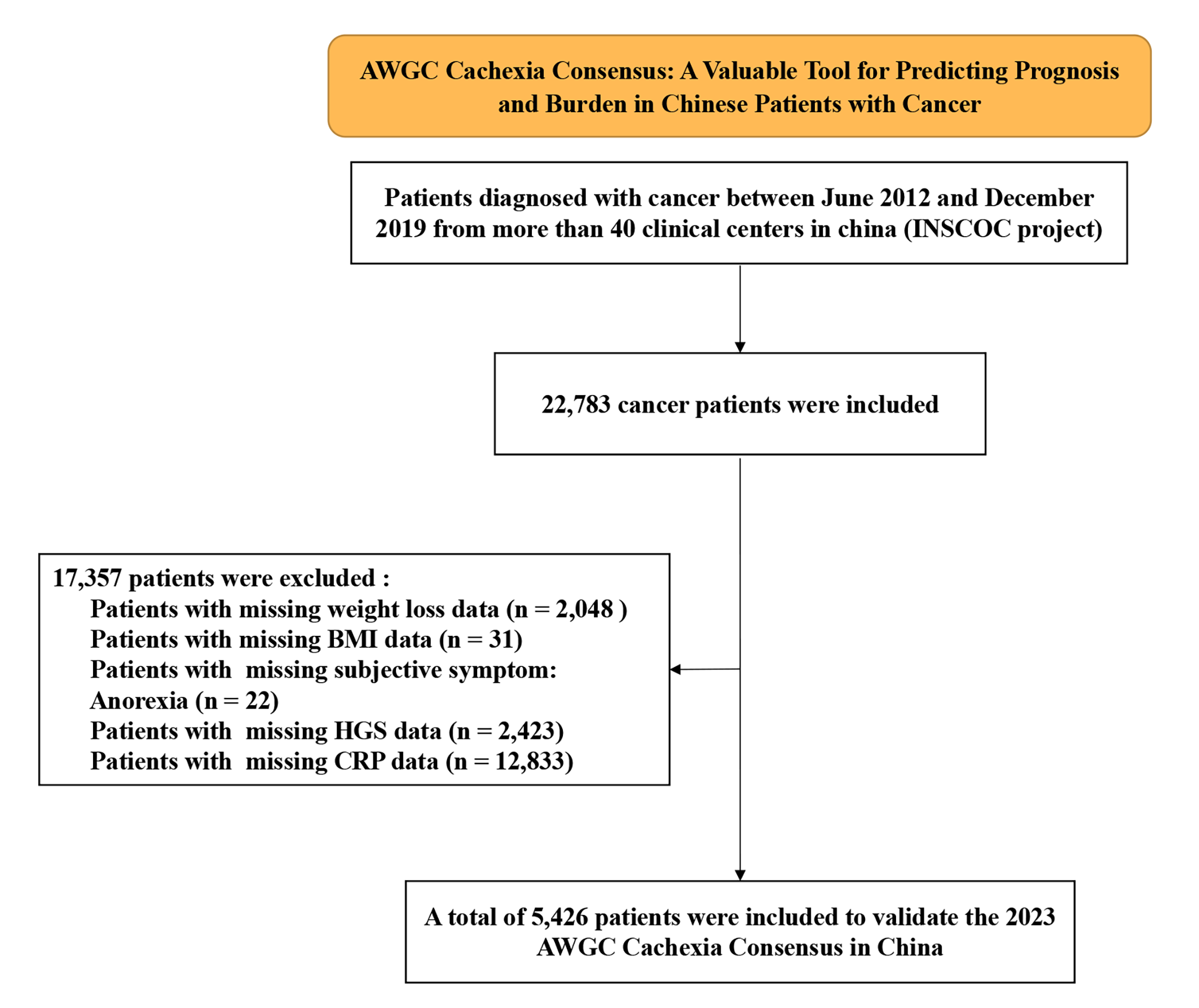
**

**Figure S3. The incidence of cachexia in different tumor types.**

**
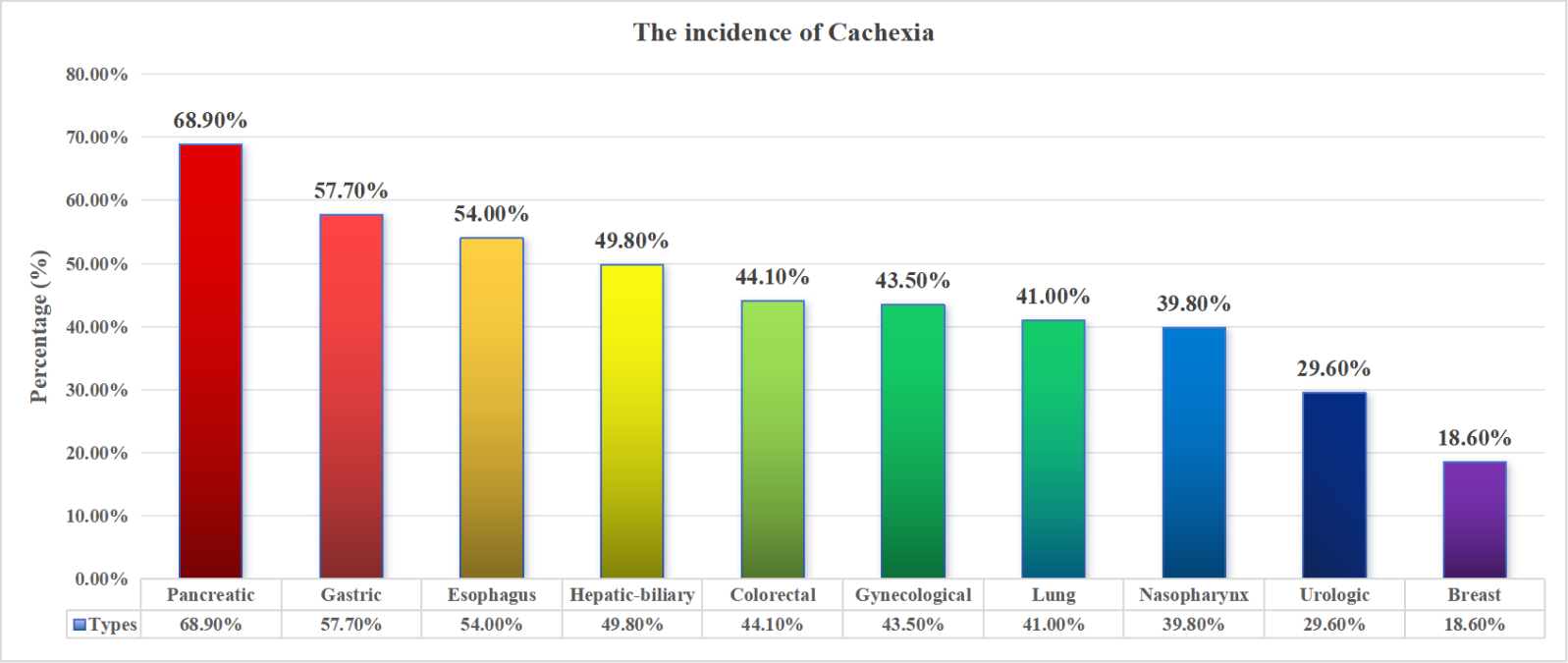
**

**Figure S4.** Kaplan-Meier curve of AWGC2023 criteria in patients with cancer at different pathological stage.

**
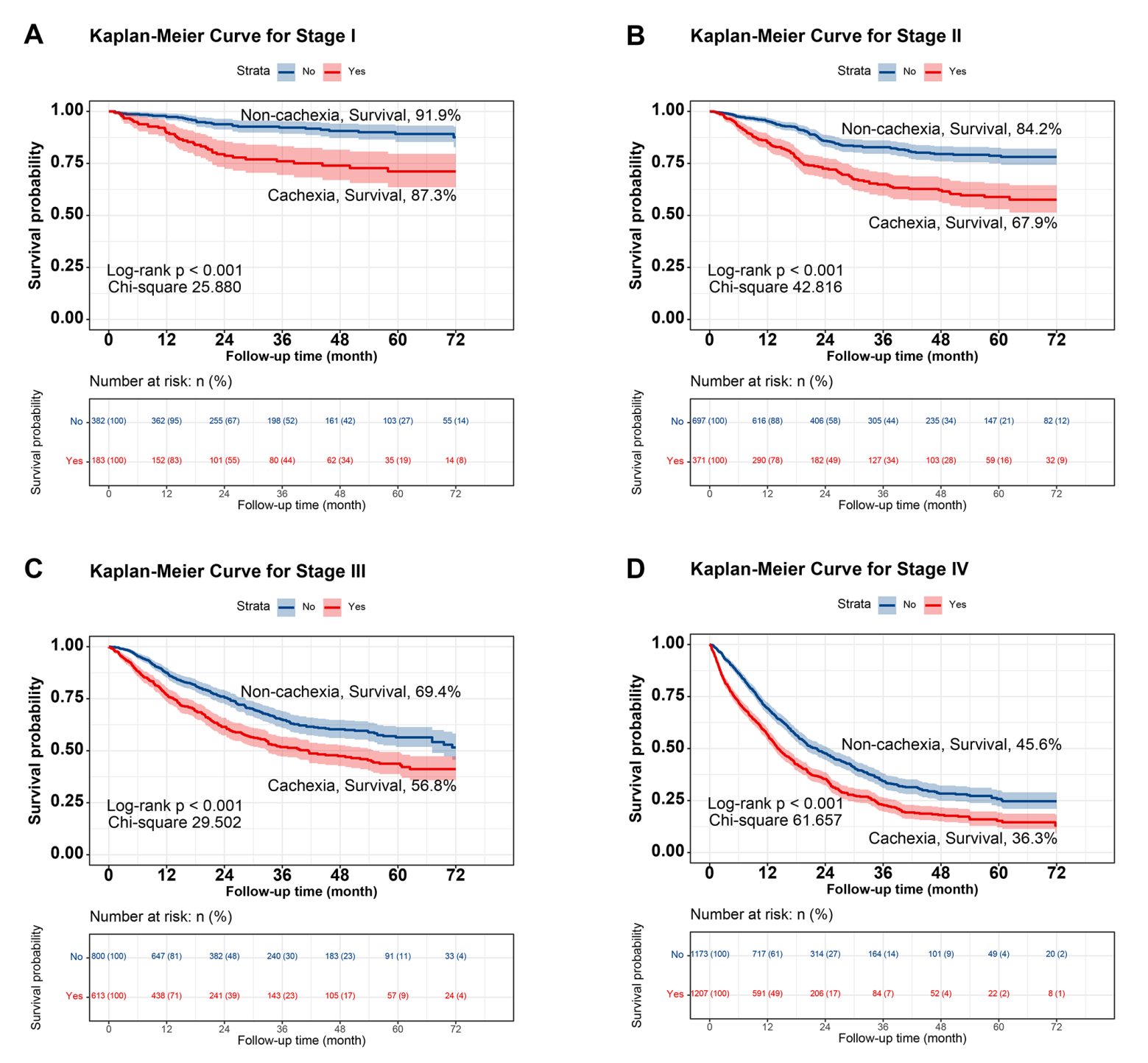
**

**Notes:** A, Stage I; B, Stage II; C, Stage III; D, Stage IV.

**Figure S5.** Subgroup Survival Forest Plot of Different Tumor Type.

**
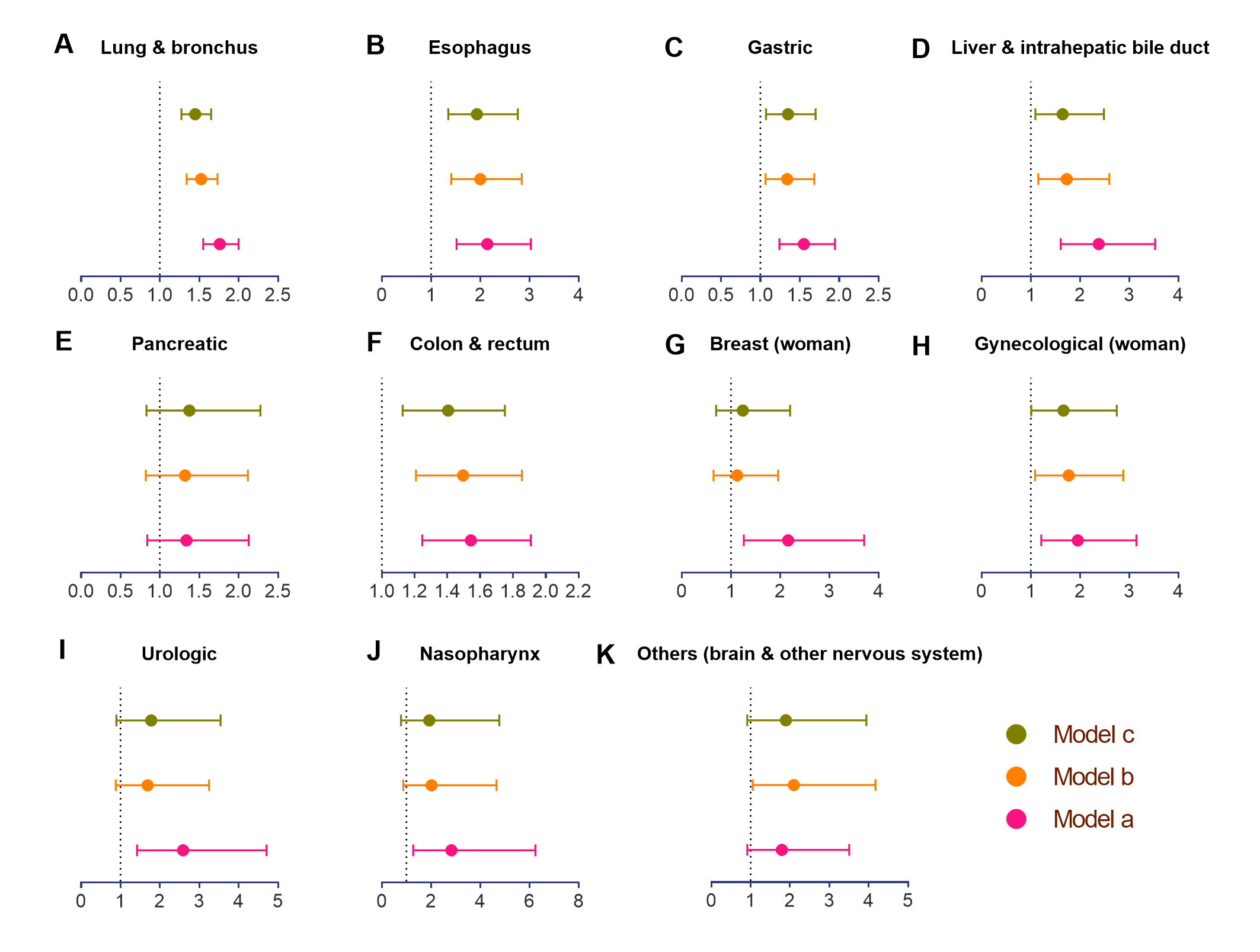
**

Notes: A, Lung &bronchus; B, Esophagus; C, Gastric; D, Live & intrahepatic bile duct; E, Pancreatic; F, Colon &rectum; G,Breast (woman); H, Gynecological (woman); I, Urologic; J, Nasopharynx; K, Others (brain & other nervous system).

Model a: No adjusted.

Model b: Adjusted for age, sex, BMI, TNM stage.

Model c: Adjusted for age, sex, BMI, TNM stage, tumor type, surgery, radiotherapy, chemotherapy, hypertension, diabetes, coronary heart disease,smoking, drinking, family history.

**Figure S6.** The intersection of diagnosed cachexia cases between the AWGC2023 criteria and the 2011 Cachexia Consensus.

**
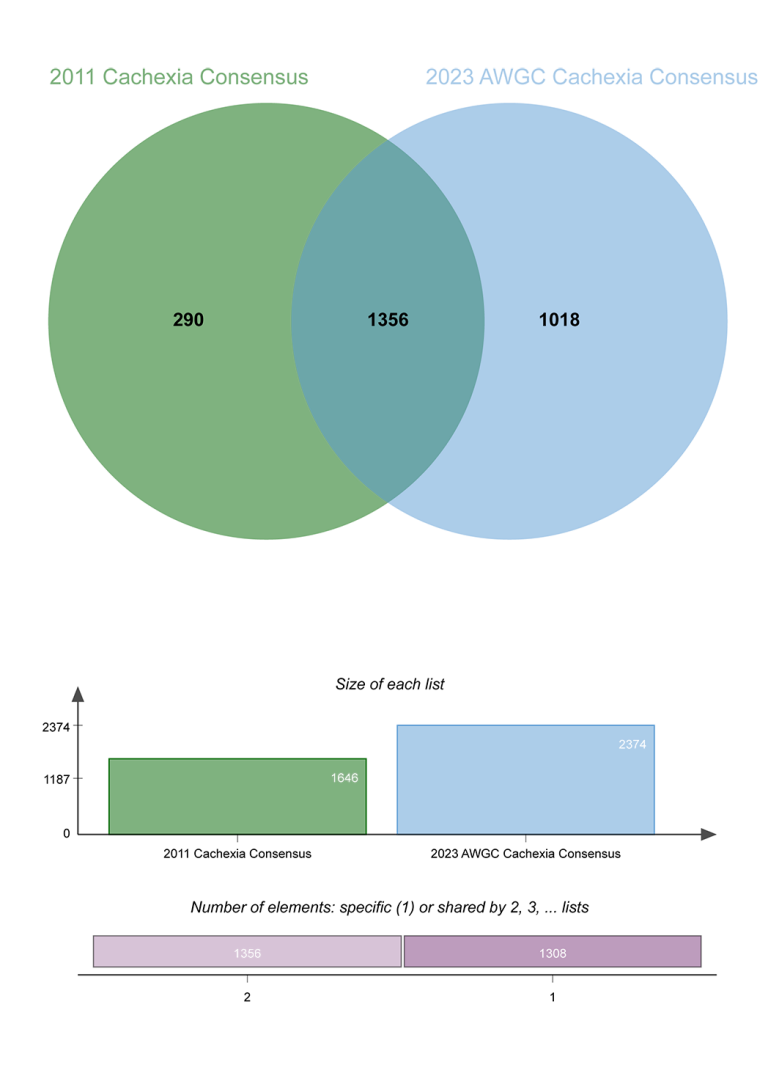
**

**Figure S7.** Venn Diagram of Various Inflammatory Markers-based Cachexia.

**
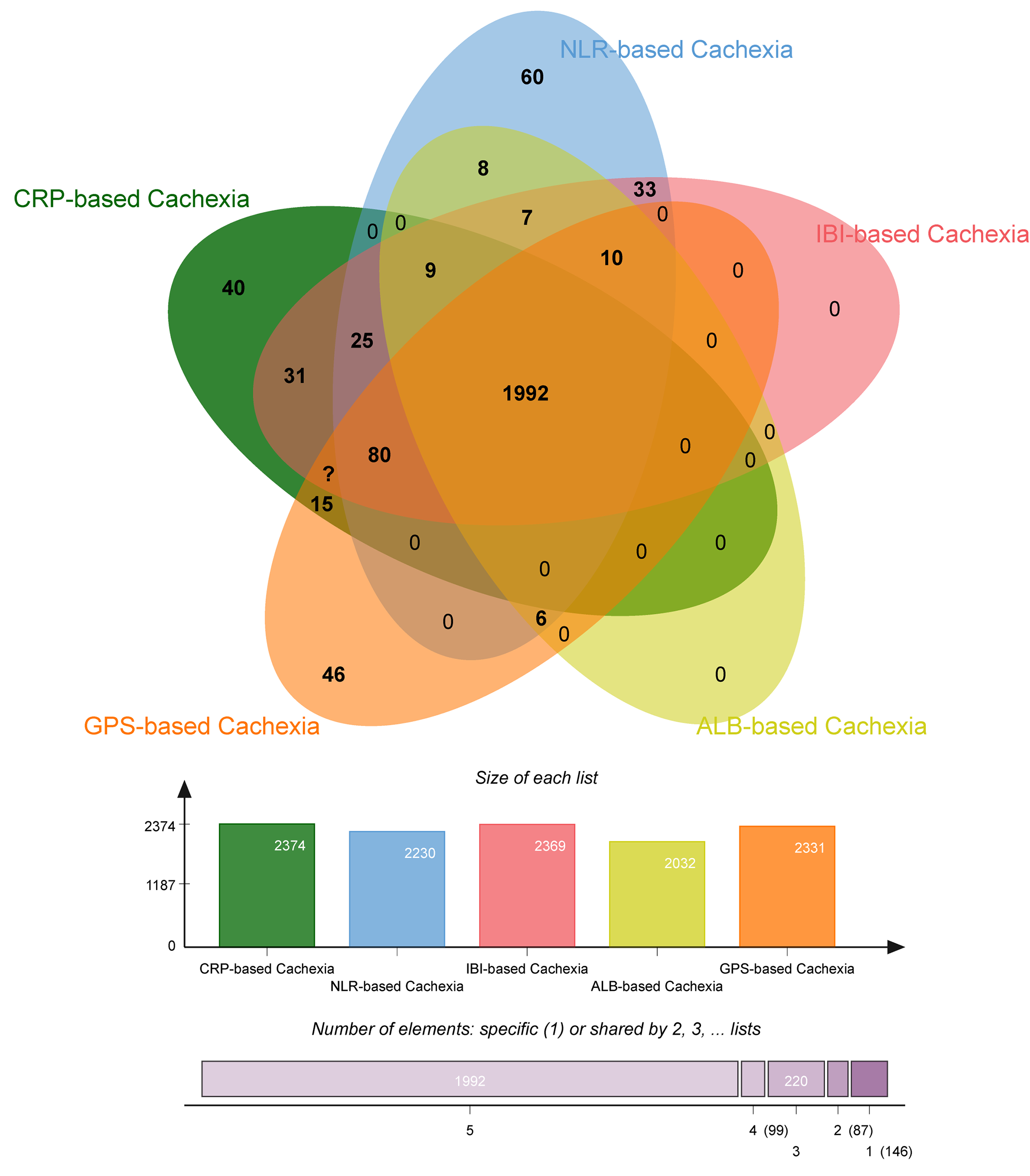
**

**Table S1.** Clinicopathological characteristics.

| **Characteristic** | Overall  (n=5426) | Cachexia | | P value |
| --- | --- | --- | --- | --- |
|  |  | No (n=3052) | Yes (n=2374) |  |
| Sex, men, n (%) | 3268 (60.2) | 1765 (57.8) | 1503 (63.3) | <0.001 |
| Age, years, mean (SD) | 59.31 (11.20) | 58.24 (10.99) | 60.69 (11.31) | <0.001 |
| BMI (median (IQR)) | 22.58 (20.31, 24.84) | 23.73 (21.92, 25.65) | 20.66 (18.94, 22.96) | <0.001 |
| Hypertension, yes, n (%) | 1059 (19.5) | 609 (20.0) | 450 (19.0) | 0.375 |
| Diabetes, yes, n (%) | 569 (10.5) | 309 (10.1) | 260 (11.0) | 0.346 |
| Coronary heart disease, yes, n (%) | 292 ( 5.4) | 155 ( 5.1) | 137 ( 5.8) | 0.289 |
| Smoking yes, n (%) | 2615 (48.2) | 1384 (45.3) | 1231 (51.9) | <0.001 |
| Drinking, yes, n (%) | 1198 (22.1) | 641 (21.0) | 557 (23.5) | 0.033 |
| Family history, yes, n (%) | 868 (16.0) | 504 (16.5) | 364 (15.3) | 0.254 |
| Tumor location, n (%) |  |  |  |  |
| Lung & bronchus | 1798 (33.1) | 1061 (34.8) | 737 (31.0) | <0.001 |
| Esophagus | 289 ( 5.3) | 133 ( 4.4) | 156 ( 6.6) |  |
| Gastric | 799 (14.7) | 338 (11.1) | 461 (19.4) |  |
| Liver & intrahepatic bile duct | 237 ( 4.4) | 119 ( 3.9) | 118 ( 5.0) |  |
| Pancreatic | 132 ( 2.4) | 41 ( 1.3) | 91 ( 3.8) |  |
| Colon & rectum | 1076 (19.8) | 601 (19.7) | 475 (20.0) |  |
| Breast (female) | 473 ( 8.7) | 385 (12.6) | 88 ( 3.7) |  |
| Gynecological (female) | 193 ( 3.6) | 109 ( 3.6) | 84 ( 3.5) |  |
| Urologic | 189 ( 3.5) | 133 ( 4.4) | 56 ( 2.4) |  |
| Nasopharynx | 113 ( 2.1) | 68 ( 2.2) | 45 ( 1.9) |  |
| Others (brain & other nervous system) | 127 ( 2.3) | 64 ( 2.1) | 63 ( 2.7) |  |
| TNM stage, n (%) |  |  |  | <0.001 |
| Stage I | 565 (10.4) | 382 (12.5) | 183 ( 7.7) |  |
| Stage II | 1068 (19.7) | 697 (22.8) | 371 (15.6) |  |
| Stage III | 1413 (26.0) | 800 (26.2) | 613 (25.8) |  |
| Stage IV | 2380 (43.9) | 1173 (38.4) | 1207 (50.8) |  |
| Surgery, yes, n (%) | 3303 (60.9) | 1967 (64.4) | 1336 (56.3) | <0.001 |
| Radiotherapy, yes, n (%) | 628 (11.6) | 376 (12.3) | 252 (10.6) | 0.057 |
| Chemotherapy, yes, n (%) | 3573 (65.8) | 2090 (68.5) | 1483 (62.5) | <0.001 |
| White blood cells (median (IQR)) | 6.06 (4.77, 7.85) | 5.86 (4.60, 7.40) | 6.40 (4.95, 8.59) | <0.001 |
| Neutrophil (mean (SD)) | 3.78 (2.64, 5.40) | 3.50 (2.53, 4.87) | 4.27 (2.88, 6.17) | <0.001 |
| Lymphocyte (mean (SD)) | 1.48 (1.09, 1.90) | 1.55 (1.16, 1.98) | 1.36 (1.00, 1.80) | <0.001 |
| Platelets (median (IQR)) | 222.00 (171.00, 281.00) | 215.00 (170.00, 267.00) | 233.00 (175.00, 300.00) | <0.001 |
| Red blood cells (median (IQR)) | 4.27 (3.83, 4.67) | 4.38 (3.99, 4.75) | 4.12 (3.64, 4.53) | <0.001 |
| Hemoglobin (median (IQR)) | 127.00 (112.00, 139.00) | 131.00 (119.00, 142.00) | 121.00 (104.00, 134.00) | <0.001 |
| ALB (median [IQR]) | 39.20 (35.50, 42.20) | 40.40 (37.40, 43.10) | 37.10 (33.60, 40.50) | <0.001 |
| CRP (median [IQR]) | 4.10 (2.50, 18.60) | 3.20 (1.50, 6.61) | 10.80 (3.23, 37.20) | <0.001 |
| NLR (median [IQR]) | 2.54 (1.71, 4.00) | 2.25 (1.58, 3.35) | 3.07 (1.95, 5.06) | <0.001 |
| HGS (median [IQR]) | 24.10 (18.10, 31.30) | 26.60 (20.50, 33.40) | 21.10 (15.33, 27.30) | <0.001 |
| WL (median [IQR]) | 0.63 (0.00, 5.80) | 0.00 (0.00, 0.79) | 5.38 (2.41, 8.81) | <0.001 |
| ECOG score (median (IQR)) | 0.00 (0.00, 1.00) | 0.00 (0.00, 1.00) | 0.00 (0.00, 1.00) | <0.001 |
| PG-SGA score (median (IQR)) | 5.00 (2.00, 8.00) | 2.00 (1.00, 5.00) | 8.00 (5.00, 11.00) | <0.001 |
| Quality of life score (median (IQR)) | 47.00 (43.00, 54.00) | 45.00 (42.00, 51.00) | 49.00 (44.00, 57.00) | <0.001 |
| Short-term outcome (mortality within 3 months), death, n (%) | 345 ( 6.4) | 96 ( 3.1) | 249 (10.5) | <0.001 |
| Long-term survival (mortality during follow up), death, n (%) | 2218 (40.9) | 1024 (33.6) | 1194 (50.3) | <0.001 |
| LOS (median (IQR)) | 10.00 (7.00, 16.00) | 10.00 (6.00, 16.00) | 11.00 (7.00, 17.00) | <0.001 |
| Expenses (median (IQR)) | 17519.40 (10008.20, 36230.90) | 16681.05 (9453.35, 32473.03) | 18812.30 (10707.50, 40883.20) | <0.001 |

**Table S2:** The sensitivity analysis by excluding the first 3, 6, 12 months mortalities of the AWGC2023 criteria and the 2011 Cachexia Consensus.

| Categories | Exclude 3 months deaths (345 patients) (HR,95%CI) | p value | Exclude 6 months deaths (658 patients) (HR,95%CI) | p value | Exclude 12 months deaths (1203 patients) (HR,95%CI) | p value |
| --- | --- | --- | --- | --- | --- | --- |
| AWGC2023 criteria | 1.376 (1.243,1.523) | <0.001 | 1.318 (1.179,1.473) | <0.001 | 1.345 (1.171,1.544) | <0.001 |
| 2011Cachexia Consensus | 1.325 (1.197,1.466) | <0.001 | 1.308 (1.17,1.463) | <0.001 | 1.335 (1.161,1.536) | <0.001 |

**Notes:** Adjusted for age, sex, tumor stage, tumor types, surgery, radiotherapy, chemotherapy, hypertension, diabetes, coronary heart disease, smoking, drinking, family history.

**Table S3:** The sensitivity analysis by excluding the first 3, 6, 12 months mortalities of the Inflammatory Markers-based Cachexia.

| Categories | Exclude 3 months deaths (345 patients) (HR,95%CI) | p value | Exclude 6 months deaths (658 patients) (HR,95%CI) | p value | Exclude 12 months deaths (1203 patients) (HR,95%CI) | p value |
| --- | --- | --- | --- | --- | --- | --- |
| NLR-based Cachexia | 1.325 (1.197,1.466) | <0.001 | 1.307 (1.168,1.462) | <0.001 | 1.311 (1.14,1.508) | <0.001 |
| IBI-based Cachexia | 1.421 (1.284,1.573) | <0.001 | 1.346 (1.205,1.505) | <0.001 | 1.374 (1.196,1.578) | <0.001 |
| ALB-based Cachexia | 1.438 (1.298,1.593) | <0.001 | 1.348 (1.205,1.509) | <0.001 | 1.363 (1.185,1.569) | <0.001 |
| GPS-based Cachexia | 1.383 (1.249,1.531) | <0.001 | 1.302 (1.165,1.456) | <0.001 | 1.303 (1.134,1.498) | <0.001 |

**Notes:** Adjusted for age, sex, tumor stage, tumor types, surgery, radiotherapy, chemotherapy, hypertension, diabetes, coronary heart disease, smoking, drinking, family history.

**Table S4.** Cox regression analysis of the Different Low BMI Cut-Off Value.

| Categories | Model a (HR,95%CI) | p value | Model b (HR,95%CI) | p value | Model c (HR,95%CI) | p value |
| --- | --- | --- | --- | --- | --- | --- |
| Cachexia (BMI<18.5) | 1.859 (1.709,2.021) | <0.001 | 1.5 (1.373,1.638) | <0.001 | 1.47 (1.344,1.607) | <0.001 |
| Cachexia(BMI<20) | 1.847 (1.699,2.007) | <0.001 | 1.473 (1.345,1.614) | <0.001 | 1.453 (1.326,1.593) | <0.001 |
| Cachexia (BMI<22) | 1.881 (1.728,2.046) | <0.001 | 1.453 (1.322,1.598) | <0.001 | 1.423 (1.293,1.566) | <0.001 |

Notes:

Model a: No adjusted.

Model b: Adjusted for age, sex, BMI, TNM stage.

Model c: Adjusted for age, sex, BMI, TNM stage, tumor type, surgery, radiotherapy, chemotherapy, hypertension, diabetes, coronary heart disease,smoking, drinking, family history.

**Table S5.** Comparative analysis of the discrimination of the Different Low BMI Cut-Off Value.

| Discrimination Ability | C-index | | |
| --- | --- | --- | --- |
|  | Difference | Difference | p value |
| Cachexia (BMI<21) | 0.587(0.576,0.598) | Ref |  |
| Cachexia (BMI<18.5) | 0.582(0.571,0.593) | -0.005(-0.010, 0.001) | 0.099 |
| Cachexia(BMI<20) | 0.584(0.573,0.595) | -0.003(-0.007, 0.001) | 0.096 |
| Cachexia (BMI<22) | 0.586(0.576,0.597) | -0.001(-0.005, 0.004) | 0.968 |

**Table S6.** Logistic regression analysis of the different cachexia criterias in predicting Short-term outcome of patients with cancer.

| Categories | Model a (HR,95%CI) | p value | Model b (HR,95%CI) | p value | Model c (HR,95%CI) | p value |
| --- | --- | --- | --- | --- | --- | --- |
| AWGC2023 criteria | 3.608 (2.833,4.596) | <0.001 | 2.248 (1.713,2.949) | <0.001 | 2.138 (1.625,2.811) | <0.001 |
| 2011 Cachexia Consensus | 2.623 (2.106,3.268) | <0.001 | 1.827 (1.437,2.322) | <0.001 | 1.777 (1.393,2.266) | <0.001 |
| NLR-based Cachexia | 3.71 (2.924,4.706) | <0.001 | 2.399 (1.835,3.138) | <0.001 | 2.271 (1.732,2.977) | <0.001 |
| IBI-based Cachexia | 3.736 (2.928,4.765) | <0.001 | 2.34 (1.781,3.074) | <0.001 | 2.203 (1.673,2.901) | <0.001 |
| ALB-based Cachexia | 3.54 (2.812,4.456) | <0.001 | 2.323 (1.788,3.018) | <0.001 | 2.209 (1.696,2.875) | <0.001 |
| GPS-based Cachexia | 3.68 (2.891,4.684) | <0.001 | 2.371 (1.806,3.112) | <0.001 | 2.246 (1.707,2.956) | <0.001 |

Notes:

Model a: No adjusted.

Model b: Adjusted for age, sex, BMI, TNM stage.

Model c: Adjusted for age, sex, BMI, TNM stage, tumor type, surgery, radiotherapy, chemotherapy, hypertension, diabetes, smoking, drinking, coronary heart disease, family history.

**Table S7.** Logistic regression analysis of the different cachexia criterias in predicting length of stay (≥14 days) of patients with cancer.

| Categories | Model a (HR,95%CI) | p value | Model b (HR,95%CI) | p value | Model c (HR,95%CI) | p value |
| --- | --- | --- | --- | --- | --- | --- |
| AWGC2023 criteria | 1.239 (1.107,1.387) | <0.001 | 1.196 (1.055,1.356) | 0.005 | 1.166 (1.025,1.326) | 0.020 |
| 2011Cachexia Consensus | 1.121 (0.993,1.265) | 0.065 | 1.082 (0.952,1.23) | 0.225 | 1.082 (0.949,1.234) | 0.238 |
| NLR-based Cachexia | 1.286 (1.148,1.441) | <0.001 | 1.25 (1.101,1.419) | <0.001 | 1.176 (1.033,1.339) | 0.014 |
| IBI-based Cachexia | 1.254 (1.12,1.404) | <0.001 | 1.215 (1.071,1.378) | 0.003 | 1.166 (1.025,1.327) | 0.020 |
| ALB-based Cachexia | 1.336 (1.191,1.498) | <0.001 | 1.297 (1.141,1.475) | <0.001 | 1.239 (1.087,1.413) | 0.001 |
| GPS-based Cachexia | 1.206 (1.077,1.35) | 0.001 | 1.157 (1.02,1.314) | 0.024 | 1.118 (0.982,1.273) | 0.091 |

Notes:

Model a: No adjusted.

Model b: Adjusted for age, sex, BMI, TNM stage.

Model c: Adjusted for age, sex, BMI, TNM stage, tumor type, surgery, radiotherapy, chemotherapy, hypertension, diabetes, coronary heart disease, smoking, drinking, family history.

**Table S8.** Logistic regression analysis of the different cachexia criterias in predicting expenses (≥20000 yuan) of patients with cancer.

| Categories | Model a (HR,95%CI) | p value | Model b (HR,95%CI) | p value | Model c (HR,95%CI) | p value |
| --- | --- | --- | --- | --- | --- | --- |
| AWGC2023 criteria | 1.207 (1.083,1.344) | <0.001 | 1.182 (1.048,1.334) | 0.007 | 1.16 (1.022,1.317) | 0.022 |
| 2011Cachexia Consensus | 1.178 (1.049,1.323) | 0.006 | 1.143 (1.01,1.292) | 0.034 | 1.112 (0.977,1.265) | 0.109 |
| NLR-based Cachexia | 1.28 (1.148,1.428) | <0.001 | 1.274 (1.128,1.439) | <0.001 | 1.187 (1.044,1.35) | 0.009 |
| IBI-based Cachexia | 1.222 (1.097,1.362) | <0.001 | 1.204 (1.067,1.359) | 0.003 | 1.162 (1.023,1.32) | 0.021 |
| ALB-based Cachexia | 1.315 (1.177,1.469) | <0.001 | 1.308 (1.156,1.48) | <0.001 | 1.241 (1.089,1.413) | 0.001 |
| GPS-based Cachexia | 1.218 (1.093,1.358) | <0.001 | 1.194 (1.058,1.348) | 0.004 | 1.156 (1.017,1.313) | 0.026 |

Notes:

Model a: No adjusted.

Model b: Adjusted for age, sex, BMI, TNM stage.

Model c: Adjusted for age, sex, BMI, TNM stage, tumor type, surgery, radiotherapy, chemotherapy, hypertension, diabetes, coronary heart disease, smoking, drinking, family history.
